# Women’s Awareness and Healthcare Provider Discussions about Zika Virus during Pregnancy, United States, 2016–2017

**DOI:** 10.3201/eid2605.190727

**Published:** 2020-05

**Authors:** Letitia Williams, Denise V. D’Angelo, Brenda Bauman, Ada C. Dieke, Sascha R. Ellington, Carrie K. Shapiro-Mendoza, Shanna Cox, Philip Hastings, Holly Shulman, Leslie Harrison, Martha Kapaya, Wanda D. Barfield, Lee Warner, PRAMS Zika State Working Group

**Affiliations:** Centers for Disease Control and Prevention, Atlanta, Georgia, USA

**Keywords:** Zika virus, PRAMS, travel advisory, healthcare provider, pregnancy, prenatal care, viruses, United States, ZIKV

## Abstract

We surveyed women with a recent live birth who resided in 16 US states and 1 city during the 2016 Zika outbreak. We found high awareness about the risk of Zika virus infection during pregnancy and about advisories to avoid travel to affected areas but moderate levels of discussions with healthcare providers.

Zika virus infection (ZIKV), transmitted primarily by the *Aedes aegypti* and *Ae. albopictus* mosquitoes, is a serious threat to pregnant women because of the risk of microcephaly and other birth defects in infants (*1*,*2*). In late 2015, an unprecedented ZIKV outbreak emerged in South America and spread rapidly into other parts of the Americas. Although the outbreak has subsided, and pregnant women residing in the United States were at risk for ZIKV infection primarily if they traveled to affected areas or had sexual contact with a partner who traveled to an affected area, information about the outbreak was publicized (*3*). In particular, travel advisories and guidance about ZIKV were published during the 2016 Zika outbreak (*4*–*6*), but the degree of awareness of ZIKV among pregnant women in the United States is unknown. We examined awareness of ZIKV, discussions about ZIKV with healthcare providers, and knowledge of travel advisories to avoid ZIKV-affected areas during pregnancy among women who delivered a live infant during the outbreak.

## The Study

The Pregnancy Risk Assessment Monitoring System (PRAMS) is a state-specific, population-based surveillance system implemented by the Centers for Disease Control and Prevention (CDC) and state and local health departments to collect information about experiences and behaviors before, during, and after pregnancy among women with a live birth. A stratified random sample is drawn from birth certificate records every month in each participating site. Women are surveyed by mail or telephone 2–6 months after a live birth. Data are weighted to account for the stratified sampling design and to adjust for differential nonresponse (*7*). We analyzed PRAMS data from 16 US states and 1 city, referred to here as sites (Alabama, Connecticut, Florida, Illinois, Maryland, Massachusetts, Missouri, New Jersey, New York, Pennsylvania, South Carolina, Tennessee, Vermont, Virginia, West Virginia, and Wisconsin, plus New York City), for women who gave birth during March 2016–February 2017.

CDC and participating sites developed supplemental questions on ZIKV in 2016 (*8*), which sites voluntarily included in their surveys. Once added to the survey, the questions were integrated into the regular PRAMS data collection system, including data processing and weighting, and were considered part of the annual dataset.

We calculated prevalence estimates and 95% CIs for 3 ZIKV-related outcomes during pregnancy, a subset of the information collected in the supplement: never having heard of ZIKV, talking to a healthcare provider about ZIKV, and having knowledge of ZIKV-related travel advisories during pregnancy. We examined these outcomes by maternal demographics, including age, race/ethnicity, education, marital status, source of payment for delivery, infant birth month, and state of residence.

We used multivariable logistic regression to assess the relationships between maternal demographics and each outcome using adjusted prevalence ratios (aPRs) and 95% CIs. We adjusted models for all demographics examined, along with factors likely to influence access to healthcare and exposure to information about ZIKV. We completed our analyses using SAS version 9.4 (https://www.sas.com) and SAS-callable SUDAAN 11.0 (https://www.rti.org) software to account for PRAMS complex survey design.

Of 12,845 women sampled from the 17 sites during the study period, 8,711 (68%) women responded. Among respondents, most women were 25–34 years of age (59.7%), were non-Hispanic white (56.9%), had more than a high school education (65.1%), were married (61.3%), and reported private insurance as a source of payment for delivery (55.8%) (data not shown). Overall, 8.8% of women had never heard of ZIKV during their recent pregnancy. These women were more likely to be <35 years of age, be non-Hispanic black or of other race, have a high school education or less, be unmarried, and report Medicaid as a source of payment for delivery than women who had heard of ZIKV (Appendix).

Focusing on the subgroup of women who had heard about ZIKV during their pregnancy, we found that more than half (58.8%) reported talking to a healthcare provider about ZIKV. Nearly two thirds (63.5%) reported that their provider initiated the conversation; the remaining third (36.4%) reported that they initiated the conversation themselves (Table 1). Compared with non-Hispanic white women, non-Hispanic black women were more likely to have talked with a healthcare provider about ZIKV (aPR 1.12, 95% CI 1.04–1.20), as were women who gave birth during September 2016–February 2017 compared with those who gave birth in earlier months (aPR 1.19, 95% CI 1.13–1.26). However, women with a high school education or less (aPR 0.91, 95% CI 0.84–0.97), women who were not married (aPR 0.92, 95% CI 0.85–0.98), and women reporting Medicaid (aPR 0.88, 95% CI 0.82–0.94) or no insurance (aPR 0.77, 95% CI 0.2–0.96) at delivery were less likely to have talked with their healthcare provider about ZIKV (Table 2).

**Table 1:** Frequency of talking with a healthcare provider about Zika virus and knowledge of CDC travel advisories among women who had heard of Zika virus during their pregnancy and delivered a live infant, 17 US PRAMS sites, March 2016–February 2017*

Maternal characteristics	Women who had heard of Zika virus during pregnancy, n = 7,920										
	Talked with a healthcare provider about Zika virus									Had knowledge of CDC travel advisory	
	Total			Provider initiated			Respondent initiated				
	No.†	% (95% CI)‡		No.†	% (95% CI)‡		No.†	% (95% CI)‡		No.†	% (95% CI)‡
Overall	4,505	58.8 (57.3–60.3)		2,889	63.5 (61.6–65.4)		1,616	36.4 (34.5–38.3)		7,204	91.9 (91.0–92.7)
Age											
<24	801	58.1 (54.5–61.7)		600	74.6 (70.2–79.0)		201	25.4 (21.0–29.8)		1,293	89.5 (87.4–91.6)
25–34	2,707	58.6 (56.7–60.6)		1,663	60.8 (58.3–63.3)		1,044	39.2 (36.7–41.7)		4,315	92.1 (91.0–93.3)
>35	993	60.3 (57.2–63.5)		622	61.2 (57.2–65.3)		371	38.8 (34.7–42.8)		1,580	93.8 (92.3–95.3)
Race/ethnicity											
White, non-Hispanic	2,314	59.1 (57.1–61.1)		1,365	58.7 (56.1–61.3)		949	41.3 (38.7–43.9)		3,794	94.1 (93.1–95.0)
Black, non-Hispanic	837	60.3 (56.4–64.1)		596	71.9 (67.3–76.5)		241	28.1 (23.5–32.7)		1,285	87.8 (85.1–90.5)
Hispanic	800	57.4 (53.6–61.1)		600	74.0 (69.7–78.4)		200	26.0 (21.6–30.3)		1,233	89.8 (87.6–92.1)
Other, non-Hispanic	525	57.9 (53.3–62.4)		313	62.2 (56.1–68.2)		212	37.8 (31.8–43.9)		838	88.6 (85.7–91.5)
Education											
High school or below	1,229	53.1 (50.1–56.1)		921	73.9 (70.3–77.5)		308	26.1 (22.5–29.7)		2,047	86.8 (84.8–88.8)
Less than high school	3,228	61.5 (59.7–63.3)		1,936	59.3 (57.0–61.6)		1,292	40.7 (38.4–43.0)		5,075	94.4 (93.5–95.2)
Marital status											
Married	2,942	61.5 (59.7–63.3)		1,742	57.9 (55.5–60.3)		1,200	42.1 (39.7–44.5)		4,655	93.9 (93.0–94.8)
Other	1,558	54.3 (51.6–57.1)		1,142	74.7 (71.6–77.9)		416	25.3 (22.1–28.4)		2,531	88.5 (86.8–90.2)
Source of payment for delivery											
Private	2,738	62.5 (60.6–64.4)		1,606	58.3 (55.8–60.8)		1,132	41.7 (39.2–44.2)		4,208	94.1 (93.1–95.0)
Medicaid	1,564	54.2 (51.6–56.8)		1,135	72.0 (68.9–75.2)		429	28.0 (24.8–31.1)		2,629	89.0 (87.5–90.6)
No insurance	113	46.7 (37.4–56.1)		91	77.5 (65.7–89.4)		22	22.5 (10.6–34.3)		206	85.4 (78.0–92.8)
Infant birth month											
2016 Mar–Aug	1,778	53.5 (51.1–55.9)		1,087	61.9 (58.7–65.2)		691	38.1 (34.8–41.3)		3,131	90.7 (89.2–92.1)
2016 Sep–2017 Feb	2,723	63.1 (61.2–65.1)		1,798	64.6 (62.3–67.0)		925	35.4 (33.0–37.7)		4,057	93.0 (92.0–94.0)

*Data aggregated for 17 sites: Alabama, Connecticut, Florida, Illinois, Maryland, Massachusetts, Missouri, New Jersey, New York, New York City, Pennsylvania, South Carolina, Tennessee, Vermont, Virginia, West Virginia, and Wisconsin. CDC, Centers for Disease Control and Prevention; PRAMS, Pregnancy Risk Assessment Monitoring System. †Unweighted. ‡Weighted.

**Table 2:** Factors associated with never having heard of Zika virus, talking with a healthcare provider, and knowledge of CDC travel advisories among women who delivered a live infant, 17 US PRAMS sites, March 2016–February 2017*

Maternal characteristic	Adjusted prevalence ratio (95% CI)†		
	Women who had never heard of Zika virus, n = 791	Women who had heard of Zika virus during pregnancy	
		Talked with a healthcare provider about Zika virus, n = 4,505	Had knowledge of CDC travel advisory, n = 7,204
Age			
<24	**1.77 (1.28–2.44)**	1.07 (0.98–1.17)	1.00 (0.97–1.02)
25–34	1.22 (0.92–1.62)	0.99 (0.93–1.06)	0.99 (0.96–1.01)
>35	Referent	Referent	Referent
Race/ethnicity			
White, non-Hispanic	Referent	Referent	Referent
Black, non-Hispanic	**1.86 (1.46–2.37)**	**1.12 (1.04–1.20)**	0.97 (0.94–1.00)
Hispanic	**0.69 (0.49–0.99)**	1.08 (1.00–1.17)	0.99 (0.97–1.02)
Other, non-Hispanic	**2.41 (1.85 –3.13)**	0.96 (0.87–1.05)	**0.92 (0.89–0.96)**
Education			
High school or below	**2.22 (1.68–2.92)**	**0.91 (0.84–0.97)**	**0.94 (0.92–0.97)**
More than high school	Referent	Referent	Referent
Marital status			
Married	Referent	Referent	Referent
Other	**1.5 (1.19–1.89)**	**0.92 (0.85–0.98)**	0.98 (0.95–1.00)
Source of payment for delivery			
Private	Referent	Referent	Referent
Medicaid	**1.45 (1.3–1.88)**	**0.88 (0.82–0.94)**	0.98 (0.95–1.00)
None	**2.46 (1.62–3.74)**	**0.77 (0.2–0.96)**	0.95 (0.88–1.02)
Infant birth month			
2016 Mar–Aug	Referent	Referent	Referent
2016 Sep–2017 Feb	**0.81 (0.67–0.98)**	**1.19 (1.13–1.26)**	1.02 (1.00–1.04)

*Data aggregated for 17 sites: Alabama, Connecticut, Florida, Illinois, Maryland, Massachusetts, Missouri, New Jersey, New York, New York City, Pennsylvania, South Carolina, Tennessee, Vermont, Virginia, West Virginia, and Wisconsin. Final sample sizes varied for each model because of missing responses. Boldface type indicates statistical significance. CDC, Centers for Disease Control and Prevention; PRAMS, Pregnancy Risk Assessment Monitoring System. †All prevalence ratio estimates adjusted for maternal age, race/ethnicity, education, marital status, delivery payment source, infant month of birth, and state of residence.

Most (91.9%) women reported knowledge of CDC travel advisories to avoid areas affected by Zika while pregnant (Table 1). Respondents reporting other non-Hispanic race versus non-Hispanic white women were less likely to have knowledge of the travel advisories (aPR 0.92, 95% CI 0.89–0.96), as were those with a high school education or less compared with women with more than a high school education (aPR 0.94, 95% CI 0.92–0.97) (Table 2).

In the adjusted analysis, women <24 years old were more likely not to have heard of ZIKV compared with women >35 years old (aPR 1.77, 95% CI 1.28–2.44), as were non-Hispanic black women compared with non-Hispanic white women (aPR 1.86, 95% CI 1.46–2.37) and non-Hispanic women of other races compared with non-Hispanic white women (aPR 2.41, 95% CI 1.85–3.13). In contrast, Hispanic women were more likely to have heard of ZIKV. Women with a high school education or less, women whose deliveries were paid for by Medicaid, and those who were uninsured at delivery were less likely to have heard of ZIKV compared with their counterparts with more than a high school education and private health insurance (Table 2).

## Conclusions

These findings highlight interactions between pregnant women and their healthcare providers in 17 sites during the height of the 2016 Zika outbreak. Awareness of ZIKV was found to be high, as was awareness of CDC travel advisories to avoid travel to Zika-affected areas during pregnancy (both >90%). This awareness was likely obtained from multiple sources, given that only half of women who heard of ZIKV reported discussing it with their healthcare provider during pregnancy. Similar results have been reported in other studies (*9*,*10*). Even though awareness was high, disparities existed, related to the smaller proportion of Hispanic and non-Hispanic black women who reported initiating discussions with providers about ZIKV. These differences suggest the opportunity to promote patient advocacy so that patients of all backgrounds feel comfortable asking about key topics if they are not raised by the provider, especially in the case of public health threats.

Our assessment is not without limitations. Data represent only women who recently gave birth to live infants in the 17 sites included in this analysis. Women seen in practices that conducted screening for travel history before a woman talked to her provider may not have reported counseling, especially if they were determined to be at low risk during the screening. Information is self-reported by the mother 2–6 months following the birth of her infant and may be subject to recall and social desirability bias.

PRAMS is the largest state- and population-based surveillance system in the United States that samples women who delivered live infants. PRAMS was augmented to collect timely data regarding patient and provider interactions related to ZIKV. Information from this analysis can fill data gaps and address the need to understand interactions between pregnant women and their healthcare providers regarding ZIKV.

Appendix More information on awareness among women and health care provider discussions about Zika virus during pregnancy, United States, 2016–2017.
