# Supplementary material for: Women’s Awareness and Healthcare Provider Discussions about Zika Virus during Pregnancy, United States, 2016–2017
Source: Emerg Infect Dis. 2020 May;26(5):998–1001. doi: 10.3201/eid2605.190727 (PMC7181904; doi:10.3201/eid2605.190727)
Supplement: Appendix — More information on awareness among women and health care provider discussions about Zika virus during pregnancy, United States, 2016–2017. [file 19-0727-Techapp-s1.pdf]

Article DOI: https://doi.org/10.3201/eid2605.190993

Women's Awareness and Healthcare Provider Discussions about Zika Virus during Pregnancy, United States, 2016–2017

Appendix

Appendix Table. Comparison of women who had not heard of Zika and those who had heard of Zika during pregnancy by maternal characteristics in 17 US sites* during March 2016–February 2017, Pregnancy Risk Assessment Monitoring System.

	Had not heard of Zika		Heard of Zika	
Maternal characteristics	No.†	% (95% CI)‡	No.†	% (95% CI)‡
Total	791	8.8 (8.0–9.7)	7920	91.2 (90.3–92.0)
Age				
<24	316	41.3 (36.3–46.4)	1487	19.6 (18.3–20.8)
25-34	384	48.1 (43.0–53.1)	4732	60.8 (59.3–62.3)
>35	91	10.6 (7.8–13.4)	1701	19.6 (18.5–20.8)
Race/ethnicity				
White, non-Hispanic	277	38.5 (33.6–43.4)	4090	58.7 (57.3–60.1)
Black, non-Hispanic	265	33.3 (28.5–38.2)	1450	14.7 (13.7–15.6)
Hispanic	111	15.8 (12.0–19.6)	1387	18.7 (17.5–19.8)
Other, non-Hispanic	135	12.4 (9.3–15.5)	951	8.0 (7.3–8.7)
Education				
High school or below	493	64.2 (59.3–69.1)	2404	32.0 (30.5–33.5)
More than high school	293	35.8 (30.9–40.7)	5440	68.0 (66.5–69.5)
Marital status				
Married	303	35.8 (31.1–40.5)	5005	63.8 (62.3–65.3)
Other	487	64.2 (59.5–68.9)	2909	36.2 (34.7–37.7)
Source of payment for delivery				
Private	228	32.1 (27.2–36.9)	4498	58.1 (56.6–59.6)
Medicaid	510	61.6 (56.6–66.6)	3026	38.7 (37.2–40.2)
No insurance	39	6.4 (3.9–8.8)	239	3.2 (2.6–3.8)
Infant birth month				
March 2016–August 2016	365	50.3 (45.3–55.3)	3487	44.4 (43.1–45.6)
September 2016–February 2017	426	49.7 (44.7–54.7)	4433	55.6 (54.4–56.9)

*Data aggregated for 17 sites: Alabama, Connecticut, Florida, Illinois, Maryland, Massachusetts, Missouri, New Jersey, New York, New York City, Pennsylvania, South Carolina, Tennessee, Vermont, Virginia, West Virginia, and Wisconsin.
†Unweighted.
‡Weighted.
